# Cumulative exposure to childhood adversity and risk of adult psychosis: a dose–response meta-analysis

**DOI:** 10.1017/S0033291725001138

**Published:** 2025-05-29

**Authors:** Aidan Flinn, Rebecca Hefferman-Clarke, Sophie Parker, Kate Allsopp, Lan Zhou, Marieke Begemann, Richard Bentall, Filippo Varese

**Affiliations:** 1 Division of Psychology and Mental Health, School of Health Sciences, Manchester Academic Health Science Centre, University of Manchester, Manchester, UK; 2 Complex Trauma and Resilience Research Unit, Greater Manchester Mental Health NHS Foundation Trust, Manchester Academic Health Science Centre, Manchester, UK; 3 Youth Mental Health Research Unit, Greater Manchester Mental Health NHS Foundation Trust, Manchester Academic Health Science Centre, Manchester, UK; 4 Cognitive Neuroscience Center, University Medical Center Groningen, University of Groningen, Groningen, The Netherlands; 5 School of Psychology, University of Sheffield, Sheffield, UK

**Keywords:** childhood adversity, dose–response, meta-analysis, psychosis, trauma

## Abstract

**Background:**

Past meta-analyses have confirmed robust associations between childhood traumatic experiences and the risk of psychosis. However, the dose–response relationship between cumulative adversity exposure and psychosis risk observed in some, but not all, previous studies in this area has not been specifically scrutinized or substantiated via recommended meta-analytic methods. This meta-analysis aimed to synthesize the available evidence on dose–response effects between childhood trauma and psychosis outcomes.

**Methods:**

PsycINFO, PubMed, EMBASE, Web of Science, CNKI, and WANFANG were searched from inception to July 2024 to identify observational studies reporting odds ratios for psychosis outcomes across multiple levels of childhood trauma exposure. Dose–response effects were extracted from eligible studies and synthesized via robust error meta-regression analyses.

**Results:**

Twenty-one studies comprising 59,975 participants were included in the meta-analysis. A significant nonlinear relationship was observed between the number of childhood adversities and the risk of future psychosis experiences (*p* for nonlinearity = .021). The pooled odds ratio for psychosis increased from 1.76 (95% confidence interval [CI]: 1.39–2.22) for 1 exposure to 6.46 (95% CI: 4.37–9.53) for 5+ exposures compared to no traumatic experience.

**Conclusions:**

This meta-analysis provides robust evidence for a dose–response relationship between cumulative childhood adversity and psychosis risk, with nonlinear patterns suggestive of an accelerating, more pronounced, risk at higher levels of trauma exposure. These findings underscore the importance of considering childhood traumatic experiences as a putative and potentially causative risk factor for psychotic experiences, as well as early prevention and intervention efforts targeting childhood adversity to reduce the risk of psychosis.

## Introduction

There is extensive research that indicates a high prevalence of potentially traumatic events in individuals who experience psychotic symptoms, noted to be as high as 80% in some samples (Hardy et al., 2016). Consequently, high levels of post-traumatic stress disorder (PTSD) and complex PTSD have also been observed, which has been replicated in nonclinical and attenuated symptom samples (de Bont et al., 2015; Ered & Ellman, 2019; Panayi et al., 2022; Peh, Rapisarda, & Lee, 2019).

Meta-analytic evidence suggests that exposure to traumatic events in childhood increases the risk of experiencing psychosis later in life (Beards et al., 2013; Pastore, de Girolamo, Tafuri, Tomasicchio, & Margari, 2020; Varese, Smeets et al., 2012; Zhou et al., 2025). Further studies indicate a more severe clinical presentation, particularly related to symptom severity, poorer functioning, and increased substance use, leading to higher service burden (Giannopoulou, Georgiades, Stefanou, Spandidos, & Rizos, 2023; Seow et al., 2023; Stanton, Denietolis, Goodwin, & Dvir, 2020). Furthermore, trauma symptoms and sequalae have been found to be particularly prevalent in patients experiencing psychosis, compared with other community samples (Rodrigues & Anderson, 2017). Particular experiences, such as dissociation and flashbacks, are involved in maintaining psychotic symptoms (Varese, Barkus, & Bentall, 2012; Williams, Bucci, Berry, & Varese, 2018). These findings suggest that the relationship is robust and well replicated, which supports theoretical viewpoints that childhood traumatic experiences are an important risk factor for psychosis (Larkin & Read, 2008).

Hill’s (1965) criteria for causality in epidemiological studies have been used to evaluate the relationship between traumatic events and psychotic experiences, and many have been subject to a wide range of empirically robust research: examining ‘temporality’ through longitudinal cohort studies (Bell, Foulds, Horwood, Mulder, & Boden, 2019; Wolke, Lereya, Fisher, Lewis, & Zammit, 2014), ‘consistency’ through meta-analytical findings (Varese, Barkus, & Bentall, 2012), and ‘specificity’ of mechanistic pathways in both clinical and nonclinical populations (Alameda et al., 2020; Bentall et al., 2014). However, less is known about whether the relationship between traumatic events and psychotic experiences follows a ‘dose–response relationship’ (also known as ‘biological gradient’). Originating from pharmacological research, this concept refers to a potential relationship between the ‘dose’ of an exposure (quantity of a medication) and a ‘response’ (likelihood of disease; Calabrese, 2016). Such patterns have been used to identify risk factors that may be causally implicated in the development of other conditions, for example, by establishing the relationships between adverse childhood experiences and depression (Tan & Mao, 2023) and between traumatic experiences and general mental health symptoms (Merckelbach, Langeland, de Vries, & Draijer, 2014).

Several research studies have identified potential dose–response relationships between traumatic events and psychotic experiences (Varese, Barkus, & Bentall, 2012). However, these associations have not been scrutinized by a previous meta-analysis of this research literature. This is in part due to the heterogeneous methodologies employed, because conventional meta-analysis methods are not suited for the identification and synthesis of dose–response patterns, and also due to the limited availability of suitable primary research studies at the time when the most influential meta-analyses of these research literatures (Varese, Barkus, & Bentall, 2012) were originally conducted. However, as time has passed, more studies have reported dose–response relationship data, necessitating the current review. This is the first meta-analysis to apply dose–response meta-analytic (DRMA) methods to determine if there is a dose–response relationship between childhood trauma and psychotic experiences, with findings that should clarify the importance of this putative risk factor for severe mental illness, with implications for primary prevention, as well as early identification and treatment for psychotic disorders.

## Methods

This preregistered review (CRD 42024549856) was completed in line with the Preferred Reporting Items for Systematic Reviews and Meta-Analysis (Page et al., 2021). The authors assert that all procedures contributing to this work comply with the ethical standards of the relevant national and institutional committees on human experimentation and with the Helsinki Declaration of 1975, as revised in 2008.

### Search strategy and study selection

Systematic searches were conducted as part of a broader review on the relationship between childhood adversity and psychosis (Zhou et al., 2025; CRD42022310002) conducted on February 9, 2023 and updated on July 7, 2024. PsycINFO, PubMed, EMBASE, Web of Science, CNKI, and WANFANG were searched using Medical Subject Headings and keywords related to Childhood Trauma and Psychosis (for further details, see Supplementary Material 1).

For the purposes of the current review, childhood traumatic experiences were used as an umbrella term for a range of adverse childhood experiences that may have a long-lasting negative impact on the physical, emotional, psychological and social development on an individual (SAMHSA, 2014). While there are debates on usage of certain terms and exact definitions in the literature (Sætren et al., 2024), the term ‘childhood trauma’ was used in the current review to indicate exposure to at least one of the following events: sexual, psychological, physical and/or emotional abuse; physical and emotional neglect; bullying; witnessing or experiencing domestic violence; familial substance misuse or incarnation; war or combat exposure; unexpected deaths or accidents; iatrogenic harm; a life threating event, or any further event that the participant(s) noted as being particular upsetting, stressful or traumatic. No specific characterization was designed for these events; however, trauma exposure measures often assess the prevalence of these experiences by an open-ended item. These were included in the current analysis to recognize the idiographic nature of potentially traumatic experiences and ensure that no experiences were missed.

Articles were included if they: (1) contained population groups that reported psychotic symptoms in those over 18, including individuals in the general population, and/or were diagnosed with a psychotic disorder; (2) measured and assessed traumatic events occurring before the age of eighteen years old and provided data on their association with psychosis risk or symptoms; (3) were published in a peer review journal; (4) were available in English, Chinese, Dutch, Italian, German, or Spanish; and (5) reported data needed for meta-analysis (i.e. odds ratios) for psychotic outcomes on individual trauma exposure counts (i.e. one exposure and two exposures). Studies were excluded if they did not report sufficient data related to traumatic experiences to investigate a potential dose–response relationship.

Study selection was completed in conjunction with the coauthors, following a two-phase procedure consisting of title and abstract screening, followed by full-text screening. This was completed independently by two researchers. Agreements were progressed to the next stage for further eligibility checking, and any discrepancies were resolved through discussions with the senior researchers. Inclusion, upon completion of full-text screening, was individually discussed with a senior researcher to reduce the risk of error. When multiple publications utilized the same sample, the article reporting the largest sample size or providing the most relevant data were included.

### Data extraction

Data extraction was completed using a purposely developed tool, covering study characteristics (i.e. participant characteristics and assessment tools) and for evaluating potential dose–response relationships, namely: trauma type experienced (e.g. physical abuse), occurrence of exposure (0 or 1), and for each ‘dose’ considered in the dose–response association examined in the paper, the corresponding effect (i.e. odds ratios) and the upper and lower limits of the associated 95% confidence interval (CI), as well as information on whether the effect was adjusted for relevant covariates (and if so, what covariates were considered). For the purposes of the current review, a dose is considered the cumulative number of distinct traumatic events that someone has experienced (e.g. someone who has experienced physical abuse, but no other traumatic experience is scored, would have a dose of 1, whereas someone who has experienced physical abuse and is bullied in school would have a dose of 2). Where studies cited another linked article for particular information (e.g. sample characteristics), this was obtained by accessing the cited article.

### Bias assessment

Risk of bias was assessed using the Johanna Briggs checklists for analytical cross-sectional, cohort, and case-control studies (JBI; Munn et al., 2020). These checklists are widely used instruments to assess the rigor of included studies using domains that included the reliability of exposure and outcome measures. The JBI for cross-sectional and cohort studies was modified by removing one item not relevant for the current review (see Supplementary Material 2). The bias rating was completed independently by two independent raters, with any discrepancies resolved via discussion with a senior researcher.

### Statistical analyses

All analyses were performed in STATA 14.0 (StataCorp LLC, Texas, US). To evaluate the potential dose–response association between childhood trauma and psychosis outcomes, the robust error meta-regression method (REMR; Xu & Doi, 2018) was used. This method was chosen for the current review due to the high prevalence of relative effects, and its ability to merge and analyze heterogeneous data. REMR uses inverse variance weighted least squares regression and cluster robust error variances to accurately synthesize dose–response data from different studies and samples, utilizing a ‘one-stage’ method that reduces the risk of over-confidence by improving error estimation and reducing bias. Whereas other dose–response methods typically involve estimating regression coefficients and variances for each study before combining them using fixed or random effect meta-analysis (Orsini, Li, Wolk, Khudyakov, & Spiegelman, 2012), REMR treats each study as a cluster and analyses the results using a cluster robust error variance (Xu & Doi, 2020). Thus, the current method aims to pool all relevant data into a single ‘dose’ for each level of exposure and provide a *p* value for nonlinearity which, if significant, highlights the presence of a of the potential dose–response relationship. For a clear relationship to be observed, a significant *p* value for nonlinearity and an effect size that increases with each exposure to a dose need to be demonstrated.

For any study that reported multiple exposures at the same level (e.g. two ORs for exposure to a single traumatic event), effect sizes were pooled using the ‘Metan’ function on STATA to produce a single variable. If two or more studies utilized the same dataset and investigated the same outcome, the article with the largest sample size was included. For the current review, it was decided to aggravate all exposure effects pertaining to psychotic experiences into a single variable, named ‘Any Psychotic Experience’. This procedure was adopted because the available data precluded more specific analyses of potential dose–response relationships for specific symptoms of psychosis. When multiple outcome variables were available, this was discussed with the senior researchers, and a decision was made on the most inclusive variable (e.g. any psychotic symptom rather than any visual hallucinations).

Sensitivity and influence analyses were conducted to identify potential influential studies and outliers, primarily through a one-study removed analysis. The primary analyses of the current review focused on the integration and examination of a potential dose–response relationship between traumatic experiences and psychosis after the exclusion of outliers (see below for details).

## Results

**Figure 1.**
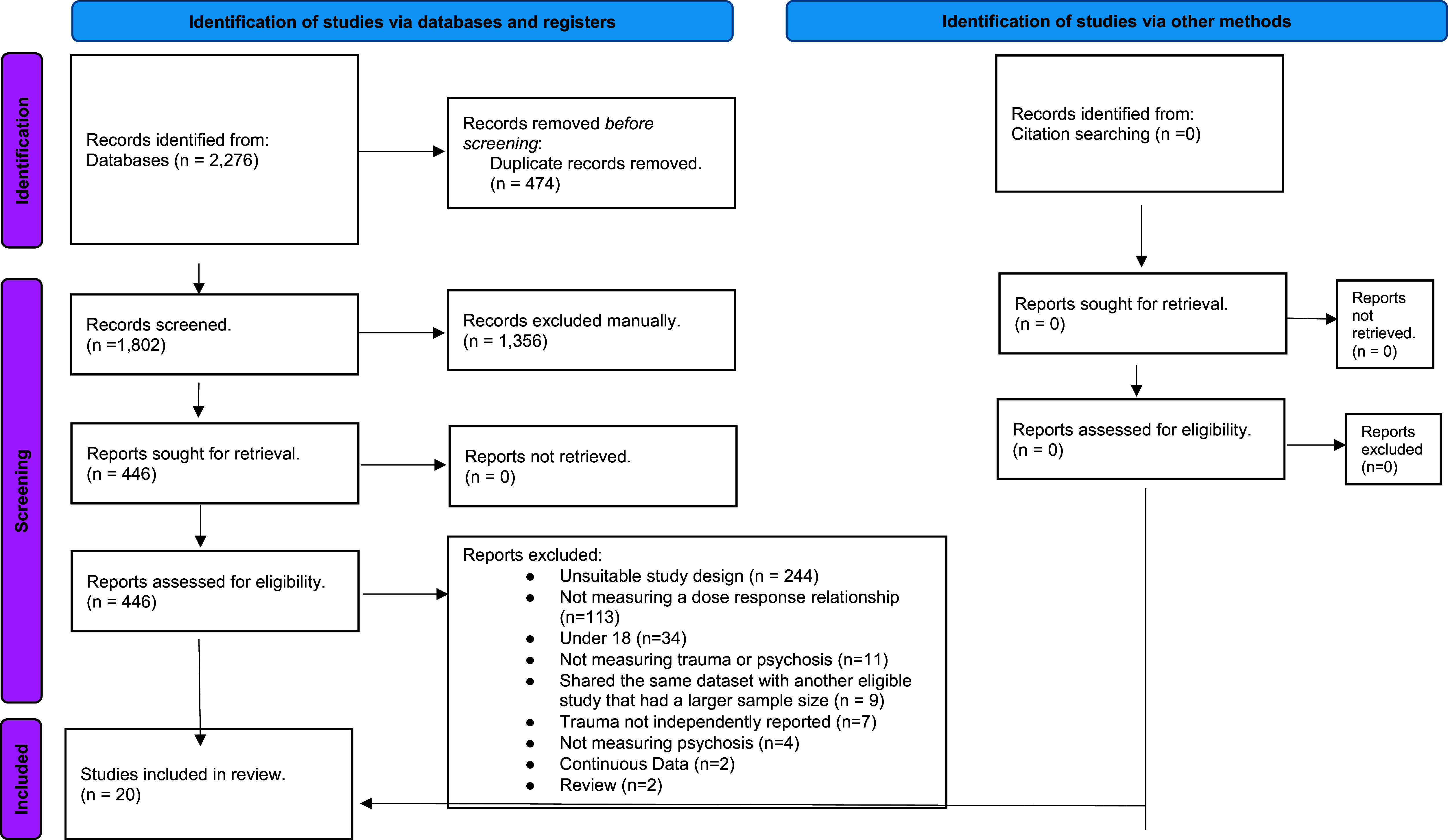
PRISMA diagram.

The search strategy resulted in 2276 total articles, which reduced to 1802 once duplicates were removed. After title and abstract screening, 446 articles remained; these articles were included in the full-text screening phase, yielding 20 studies included in the final analysis (see Figure 1). This is in contrast to the 22 studies included in the original systematic review. The final analysis after sensitivity checks comprised 11 cross-sectional studies (with a total of 52,352 participants) and 9 case-control studies (with a total of 7,623 participants, consisting of 3,324 cases and 4,299 controls), leading to a final sample size of 59,975. Participants’ mean ages ranged from 20.6 to 57 years, and a gender breakdown between 11.9% and 100% female. The included studies were spilt between clinical (*n* = 9) and nonclinical (*n* = 11) samples. Psychosis was predominantly assessed using semi-structured interview tools, such as the CIDI, SCID, MINI, or PANSS (*n* = 13); however, additional studies utilized diagnostic codes (*n* = 6) or self-report measures (*n* = 1). Exposure to traumatic experiences was assessed with varied self-report measures (*n* = 15), semi-structured interviews (*n* = 4), and service records (*n* = 1). Further characteristics of the included studies are summarized in Table 1. Further characteristics of the included studies are summarized in Table 1.

**Table 1.** Characteristics of included studies reporting dose–response relationships between cumulative traumatic events and psychotic experiences

Authors	Total sample size (type)	Mean age (SD)	Gender (%)	N cases	N controls	Trauma type measured	Psychotic experience measured	Trauma measure	Psychosis measure
*Cross-sectional studies*									
Abajobir et al. (2017)	3752 (clinical)	20.6 (SD not reported)	Not reported	N/A	N/A	Maltreatment	Any DSM-IV Psychosis	Service records (I.e. CPS)	CIDI-Auto
Bentall, Wickham, Shevlin and Varese (2012)	7353 (nonclinical)	Not reported	Not reported	N/A	N/A	Sexual, physical abuse, bullying, parental Separation and institutional care	Auditory verbal hallucinations And paranoid ideation	Self-report measure	PSQ
Croft et al. (2019)	412 (nonclinical)	Not reported	Not reported	N/A	N/A	Domestic violence, physical, emotional & sexual abuse, bullying and emotional neglect	Any psychotic experience	Self-report measure	PLIKSi
Janssen et al. (2004)	4045 (nonclinical)	41.4 (11.8)	2130 Women (52.7%)	N/A	N/A	Emotional neglect, psychological, physical and sexual abuse	Any psychotic experience	Semi-structured interview	CIDI 1.1
Kennedy, Tripodi, Pettus-Davis, and Ayers (2016)	230 (nonclinical)	33.7 (9.9)	230 Women (100%)	N/A	N/A	Physical and sexual abuse	Positive symptoms of psychosis	CTQ	MINI
Liu et al. (2022)	4441 (nonclinical)	43.4 (SD not reported)	2189 Women (51.5%)	N/A	N/A	Physical, emotional, and sexual abuse; physical and emotional neglect; Domestic violence; Parental death or divorce; Incarcerated family members, familial substance abuse or Mental illness and childhood bullying	Positive symptoms of Psychosis	ACE-Q	CIDI 3.0
McGrath et al. (2017)	1661 (nonclinical)	Not reported	Not reported	N/A	N/A	Maladaptive family functioning and Childhood Adversities	Any psychotic experiences	WMH Surveys	CIDI
Morgan et al. (2014)	1680 (nonclinical)	39 (16.9)	Not reported	N/A	N/A	Unexpected death, relationship breakdown, Financial concerns, accidents, witnessed violence, victim of crime, War exposure, physical and sexual abuse	Any Psychotic experiences	Self-Report measures	PSQ
Shevlin, Dorahy, and Adamson (2007)	5877 (nonclinical)	32.02 (10.59)	51.9% Women	N/A	N/A	Childhood neglect, Physical Abuse, Rape, Molestation	Auditory, tactile and Visual Hallucinations	adapted PTSD module from CIDI 3.0	Diagnostic code
Shevlin et al. (2011)	5594 (nonclinical)	44.35 (17.27)	58% Women	N/A	N/A	Sexual Trauma or Domestic violence	Visual or auditory Hallucinations	Adapted PTSD module from CIDI 3.0	CIDI 3.0
Whitfield, Dube, Felitti, and Anda (2005)	17,307 (clinical)	57 (15.3)	9,367 (54%) women	N/A	N/A	Emotional, physical, sexual, domestic abuse. household Substance abuse, Mental Illness, separation/divorce, incarceration.	Any Hallucinations	adapted CTS	Self-report measure
*Case control studies*									
Aas et al. (2023a)	1074 (clinical)	Case– 31.8 (10.9), Control– 38.2 (13.4)	Case– 148 (38.5%) Control– 366 (53.1%) Women	384	690	Physical, emotional, sexual Abuse, physical neglect and emotional neglect	First episode of psychosis	CTQ	Diagnostic code
Aas et al. (2023b)	908 (clinical)	Case– 29.5 (9.4), Control– 31.1 (7.6)	Case– 178 (38.6%) Control– 199 (44.5%) Women	461	447	Physical abuse, emotional abuse, sexual abuse, physical neglect and emotional neglect	Any schizophrenia diagnosis	CTQ	SCID–1
Alkema et al. (2024)	1916 (clinical)	Not reported	Not reported	577	1339	Abuse and neglect	Any schizophrenia diagnosis	CTQ-SF	CASH, SCAN, SCID–1
Arranz et al. (2018)	207 (clinical)	Case– 22 (SD not reported) Control– 23 (SD not reported)	Case– 52 (35.6%) Control– 29 (47.5%) Women	146	61	Emotional, Physical and Sexual abuse and Emotional and physical neglect	First episode of psychosis	CTQ-SF	Diagnostic code
Fisher et al. (2010)	428 (clinical)	Case– 31 (11.3) Control– 39 (12.7)	Case– 84 (46.2%) Control– 143 (58.1%) Women	182	246	Physical abuse, familial neglect, sexual abuse and lack of support figure.	Any psychotic disorder	CECA.Q	Diagnostic Code
Mall et al. (2020)	2097 (clinical)	36.1 (9.13)	11.9% Women	1008	1089	Physical abuse, emotional Abuse, Sexual abuse, physical neglect and emotional neglect	Diagnosis of schizophrenia or schizoaffective disorder	CTQ	SCID–1
Schalinski et al. (2019)	250 (nonclinical)	28.6 (8.8)	57 (31.7%) Women	180	70	Serious injury, a life threat, sexual assault or witnessing these events	Any psychotic experience	LEC	PANSS
Trauelsen et al. (2015)	202 (clinical)	Case– 22.5 (SD not reported) Control– 22 (SD not reported)	Case– 26 (53%) Control– 26 (53%)	101	101	Physical abuse, emotional abuse, sexual abuse, physical neglect and emotional neglect	Any schizophrenia diagnosis	CTQ	Diagnostic Code
Trotta et al. (2016)	541 (clinical)	Case– 28.9 (9.3) Control– 29.2 (9.9)	Case– 113 (39.6%) Control– 119 (46.5%)	285	256	Physical abuse, sexual abuse, separation or death of a parent	First episode psychosis	CECA.Q	Diagnostic code

No studies were removed after bias ratings, with most studies fulfilling most of the criteria (See Supplementary Materials 3 and 4). Sensitivity and influence analysis indicated that a single study, Whitfield et al. (2005), exerted undue influence on the findings of the meta-analysis, by decreasing the overall effect size. This study included a larger range of ‘doses’ (up to seven) compared to other included studies, which alongside it is large sample size, caused undue influence. Consequently, the study was excluded from the primary analysis. The removal of this article did not alter any further effect sizes in a significant way (see Supplementary Material 5). Other sensitivity analyses aimed to assess the impact of including ORs that were combined into a single effect capturing all relevant psychosis outcomes ahead of the DRMA; the exclusion of these effects did not substantially influence the DRMA findings (See Supplementary Material 6).

**Figure 2:**
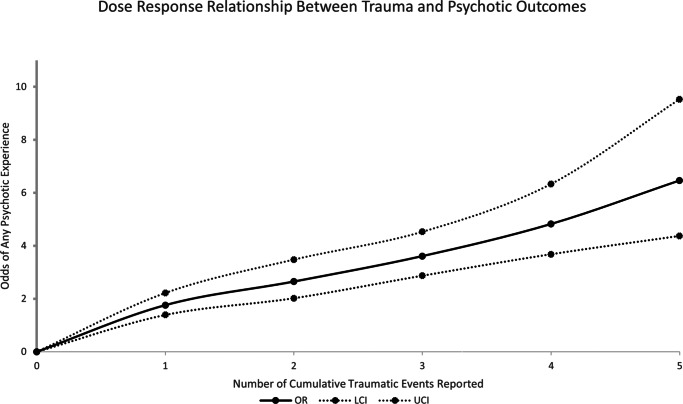
Dose–response analysis between cumulative traumatic event(s) and psychotic experiences with the solid line and the long-dashed line represents the odds ratio and 95% confidence intervals.

The results of the DRMA are presented in Table 2, with defined doses, number of exposures to cumulative traumatic events, ranging from 1 exposure to 5. A significant nonlinear relationship (*p* for nonlinearity = .021) was observed between the number of cumulative traumatic event(s) and risk of any reported psychotic experience. Risk was shown to significantly increase with each cumulative event reported, ranging from an OR of 1.76 (95% CI = 1.39–2.22) for a single exposure to an OR of 6.46 (95% CI = 4.37–9.53) for 5+ exposures. This indicates a clear dose–response relationship with a more pronounced risk at higher reported cumulative events (See Figure 2).

**Table 2.** Results of the dose–response meta-analysis, highlighting the risk of psychotic experiences with each cumulative traumatic event reported

Number of cumulative traumatic events reported—‘Doses’	OR	95% CI
0	1.00	(1.00–1.00)
1	1.76	(1.39–2.22)
2	2.65	(2.02–3.48)
3	3.61	(2.87–4.53)
4	4.82	(3.68–6.33)
5+	6.46	(4.37–9.53)

## Discussion

The present review is the first, comprehensive meta-analysis to test for a significant dose–response relationship between childhood traumatic events and increased risk of experiencing psychosis. A comprehensive search strategy was used to ensure that all eligible studies were identified and included.

Our findings indicated that each additional exposure to traumatic events is associated with a corresponding increase in the strength of the association between trauma and psychosis outcomes, highlighting the clinical significance of cumulative exposures in conveying heightened psychosis risk in the included studies. These findings have important implications for corroborating and understanding the relationship between childhood trauma and the increased risk of developing psychosis.

Current findings are consistent with meta-analytical literature that have observed an association between traumatic exposure and increased risk of experiencing psychosis, but significantly expands on these findings by providing clearer evidence for the presence of a ‘biological gradient’, otherwise known as a dose response interaction, within this relationship, that is, one of Hill’s (1965) criteria for determining potentially causal associations between health outcomes and putative risk factors (Beards et al., 2013; Pastore et al., 2020; Varese, Barkus, & Bentall, 2012; Zhou et al., 2025). Moreover, these findings are consistent with the key theoretical models that frame exposure to psychosocial stress as a critical element of psychosis-vulnerability, including the diathesis-stress model (Zubin & Spring, 1977), the social defeat hypothesis of psychosis (Selten & Cantor-Graae, 2007; Selten, Van Der Ven, Rutten, & Cantor-Graae, 2013), and cognitive models that assume that psychological and emotional responses to trauma and adversity represent key mechanisms in the development and maintenance of psychotic symptoms (Garety, Kuipers, Fowler, Freeman, & Bebbington, 2001; Hardy, 2017; Larkin & Morrison, 2007).

These findings add to the growing empirical research that has identified a number of psychological processes that may mediate the association between childhood adversity and psychosis, which includes dissociation, maladaptive cognitive factors, PTSD symptoms among others (Alameda et al., 2020; Sideli et al., 2020; Williams et al., 2018). They also indicate the potential benefit of further research that could establish whether the impact of childhood traumatic experiences on these processes is also dose and exposure dependents.

Considering that childhood traumatic experiences have been indicated as being a transdiagnostic risk factor for psychopathology (Hogg et al., 2023), it is worth noting that some mechanisms may be shared between certain mental health responses to traumatic events in childhood, potentially accounting for their comorbidity, whereas others may be specific to certain outcomes. This finding is highlighted by a previous DRMA analysis, which indicated exposure to adverse childhood experiences increased the risk of depression in a significant, nonlinear, dose–response trend similar to that observed here (Tan & Mao, 2023), strengthening the need for further research in this area.

Several methodological limitations should be acknowledged when considering these results. First, there was a significant variance in trauma type(s) and the nature of psychotic experience assessed in the included studies. This is primarily attributed to the various assessment measures being used to establish the presence of trauma and psychotic experiences. Table 1 highlights these disparities, with some studies focusing on individual experiences (e.g. childhood physical abuse), and others taking a wider berth (e.g. all forms of childhood abuse). These papers also mainly considered a certain definition of trauma, which excluded some experiences (such as racial abuse, poverty, etc.). This can limit findings, as a substantial proportion of the population in the present analysis had not been assessed for certain forms of trauma or psychotic experiences. Combining this with the recall effects that are present when assessing, retrospectively, for childhood trauma (Krayem, Hawila, Al Barathie, & Karam, 2021) means that the effect reported may well be more pronounced than demonstrated in our analysis.

In an attempt to address this issue, the present analysis aggregated experiences into two categories of ‘trauma exposure’ and ‘risk of psychosis.’ However, as a consequence of this decision, it is possible that some specificity is lost. This highlights the need for further dose–response research to focus on specific psychotic and traumatic experiences. This may require further data collection as such specific analyses were not possible with the data available for the current review.

Furthermore, the lack of longitudinal studies in the final review highlights a limitation in the current literature on the potential dose–response relationship. This indicates the need for more consistent methodology, study design, and assessment tools between studies and minimize the heterogeneity of findings.

The current review was unable to estimate precisely the probable statistical heterogeneity in study findings due to the REMR analytic method utilized. The rationale paper (Xu & Doi, 2018) suggests that heterogeneity analyses are not required because of the relevant data being aggregated to a single ‘dose’ in the analysis. We made efforts to establish heterogeneity utilizing an alternative DRMA method (Orsini et al., 2012), but this was unsuccessful, as most studies did not include the relevant data required for this purpose. Nonetheless, in light of the clinical and methodological heterogeneity evident in the included studies, is highly likely that heterogeneity is present in the effects included in this review. In order to address this for future reviews, future studies attempting to investigate the potential dose response relationship between traumatic events and psychosis require full reporting of all statistical information required to assess heterogeneity, such as the exact number(s) of each participant in the case and control groups for each exposure level. It would also be beneficial to develop meta-analytic techniques for assessing dose–response relationships that use individual participant data rather than data aggregated within individual studies.

Finally, based on the current findings, it is not possible to rule out the influence of other factors when considering an individual’s risk of psychotic experiences. It has been well established that other influences, including but not limited to genetics and substance use (Hartz et al., 2014; Howes, McCutcheon, Owen, & Murray, 2017), can impact the risk of psychotic experiences. While the findings of the present review highlight the role of trauma, psychotic disorders consist of complex presentations that are likely brought about and maintained by multiple factors, limiting our ability to assert confidently that cumulative exposure to traumatic experiences is causal to an increase in psychosis risk.

The present findings have important clinical implications. First, the positive dose–response relationship between childhood traumatic events and psychotic outcomes supports the need for the routine offer of trauma assessments for those who are at risk of or experiencing psychosis. Previous research has indicated that this approach is valued by participants if it is conducted sensitively (Read, 2007). Furthermore, it is particularly important, from a public health perspective, to address and offer trauma-informed support at the earliest opportunity, especially for children and adolescents who may have experienced early life stressors, and implement measures for primary prevention of adverse childhood events.

The results further support the growing evidence base for offering trauma-focused psychological interventions for people with psychosis. Recent clinical trials and systematic reviews have demonstrated the safety, acceptability, and efficacy of trauma therapies in people with psychosis (Hardy et al., 2024; Varese et al., 2024). While the efficacy of these interventions is particularly pronounced for comorbid post-traumatic symptoms (Brand, McEnery, Rossell, Bendall, & Thomas, 2018). Recent evidence indicates that psychotic symptoms may also improve following trauma-focused therapy and that trauma-focused interventions may be a valued treatment option also for individuals who do not have a formal diagnosis of comorbid PTSD (Burger et al., 2024; Paulik, Steel, & Arntz, 2019; van den Berg & van der Gaag, 2012). Furthermore, since many individuals who are assessed at clinically elevated risk for future psychosis have a history of trauma (Mayo et al., 2017; Peh et al., 2019), trauma-focused interventions may be integrated into indicated prevention services for preventing or delaying the onset of psychosis. Indeed, early evaluation trials suggest that trauma-focused treatments can reduce the attenuated symptoms of psychosis in at risk individuals (Strelchuk et al., 2024; Zhao et al., 2023; Varese et al., in press), but larger studies need to be completed to replicate this finding.

In conclusion, the current review found evidence of a significant nonlinear dose–response relationship between cumulative childhood traumatic events and psychotic experiences, with a more pronounced risk at higher reported cumulative events. This finding provides further evidence on the etiology and maintenance of psychotic disorders and restates the need to investigate the relationship between childhood traumatic events and psychosis risk, including the development and delivery of appropriate interventions for treating and preventing psychosis in people who have experienced trauma in their lives.

## Supporting information

Flinn et al. supplementary material Flinn et al. supplementary material

## Supplementary material

The supplementary material for this article can be found at http://doi.org/10.1017/S0033291725001138.
