# Supplementary material for: Cumulative exposure to childhood adversity and risk of adult psychosis: a dose–response meta-analysis
Source: Psychol Med. 2025 May 29;55:e162. doi: 10.1017/S0033291725001138 (PMC12150334; doi:10.1017/S0033291725001138)
Supplement: Flinn et al. supplementary material [file S0033291725001138sup001.docx]

Supplementary Material One- Search Strategy

("Psychotic Disorders"[Mesh] OR psychos*[tiab] OR psychoti*[tiab] OR schizo*[Title/Abstract] OR hallucinat*[Title/Abstract] OR delusion*[Title/Abstract] OR paranoi*[Title/Abstract])

AND ("Child"[Mesh] OR "Adolescent"[Mesh] OR child*[Title/Abstract] OR adolescen*[Title/Abstract] OR pediatr*[Title/Abstract] OR paediatr*[Title/Abstract] OR youth[Title/Abstract] OR teen*[Title/Abstract] OR school*[Title/Abstract] OR preschool*[Title/Abstract] OR preadolescen*[Title/Abstract] OR kids[Title/Abstract] OR “young people”[Title/Abstract] OR “younger people” [Title/Abstract] OR “early life”[Title/Abstract] OR “early in life”[Title/Abstract] OR “early age”[Title/Abstract] OR “younger age”[Title/Abstract] OR “young age”[Title/Abstract])

AND (Adverse Childhood Experiences[MeSH Terms] OR "Adult Survivors of Child Abuse"[Mesh] OR "Child Abuse"[Mesh] OR advers*[Title/Abstract] OR trauma*[Title/Abstract] OR “Early Life Stress”[Title/Abstract] OR “Early Stressful Event*”[Title/Abstract] OR abus*[Title/Abstract] OR “sexual harassment*”[Title/Abstract] OR rape[Title/Abstract] OR psychotrauma*[Title/Abstract] OR neglect*[tiab] OR maltreat*[Title/Abstract] OR victim*[Title/Abstract] OR abandon*[Title/Abstract] OR foster*[Title/Abstract] OR adopt*[Title/Abstract] OR violen*[Title/Abstract] OR bully[Title/Abstract] OR bullied[Title/Abstract] OR “parental loss*”[Title/Abstract] OR “parental death”[Title/Abstract] OR divorce*[Title/Abstract] OR “parental discord*”[Title/Abstract] OR separat*[Title/Abstract] OR discriminat*[Title/Abstract] OR “family discord*”[Title/Abstract] OR “parental communication deviance*”[Title/Abstract] OR “expressed emotion*”[Title/Abstract]) NOT ("animals"[MeSH] NOT "humans"[MeSH]) NOT "Review" [Publication Type] NOT "Congress" [Publication Type]

Supplementary Material Two- Modified JBI Checklist used for cross sectional studies

JBI Critical Appraisal Checklist for
analytical cross sectional studies

Reviewer ______________________________________ Date_______________________________

Author_______________________________________ Year_________ Record Number_________

|  | Yes | No | Unclear | Not applicable |
| --- | --- | --- | --- | --- |
| 1. Were the criteria for inclusion in the sample clearly defined (yes for random probability or quota sampling; no for convenience sampling)? | □ | □ | □ | □ |
| 1. Were the study subjects and the setting described in detail (setting – country, region; if from services which services; demographics of sample described) and was the sample representative? Online samples with demographics are unclear (because we don’t know where they came from), | □ | □ | □ | □ |
| 1. Was the exposure measured in a valid and reliable way? (How was the trauma measured?) Yes for structured interview or questionnaire; unclear for clinical records; no for clinician report. (Note – if composite measure used, then Yes if the components are reliable measures). | □ | □ | □ | □ |
| 1. Were confounding factors identified? (Did they measure age, sex, ethnicity, education, SES – yes for any 3; unclear for 1-2, no for 0) | □ | □ | □ | □ |
| 1. Were strategies to deal with confounding factors stated? (Did the statistical analyses take into account at least some of these factors? Sex necessary; yes if sex + 1 other; unclear if only sex; no if none into account) | □ | □ | □ | □ |
| 1. Were the outcomes measured in a valid and reliable way? (How was psychosis measured – yes for any reliable interview, eg PANSS, SCID, SCAN, or questionnaire, eg. CAPE or PDI; unclear for made up instrument; no for clinical records or clinician judgment). | □ | □ | □ | □ |
| 1. Was appropriate statistical analysis used? (Yes for correlation or regression to tests association between trauma severity and psychosis severity; or for t-test or ANOVA to compare high vs low trauma groups; no for subjective impression) | □ | □ | □ | □ |

Overall appraisal: Include □ Exclude □ Seek further info □

Supplementary Material Three- Bias rating for Cross Sectional Studies

| **Author** | **Year** | **Question 1** | **Question 2** | **Question 3** | **Question 4** | **Question 5** | **Question 6** | **Question 7** | **Score** |
| --- | --- | --- | --- | --- | --- | --- | --- | --- | --- |
| Abajobir et al. | 2017 | Yes | Yes | Unclear | Yes | Yes | Yes | Yes | 6 |
| Bentall et al. | 2012 | Yes | Yes | Yes | Yes | Yes | Yes | Yes | 7 |
| Croft et al. | 2019 | Yes | Yes | Yes | Yes | Yes | Yes | Yes | 7 |
| Jansen et al. | 2004 | Yes | Yes | Unclear | Yes | Yes | Yes | Yes | 7 |
| Kennedy et al. | 2015 | Yes | Yes | Yes | Unclear | No | Yes | Yes | 5 |
| Liu et al. | 2022 | Yes | Yes | Yes | Yes | Yes | Yes | Yes | 7 |
| McGrath et al. | 2017 | Yes | Yes | Yes | Unclear | Yes | Yes | Yes | 6 |
| Morgan et al. | 2014 | Yes | Yes | Yes | Yes | Yes | Yes | Yes | 7 |
| Shevlin et al. | 2010 | Yes | Yes | Yes | Yes | Yes | Yes | Yes | 7 |
| Shevlin et al. | 2007 | Yes | Yes | Yes | Yes | Yes | Yes | Yes | 7 |
| Whitfield et al. | 2005 | Yes | Yes | Yes | Yes | Yes | No | Yes | 6 |

Supplementary Material Four- Bias rating for Case Control Studies

| **Author** | **Year** | **Question 1** | **Question 2** | **Question 3** | **Question 4** | **Question 5** | **Question 6** | **Question 7** | **Question 8** | **Question 9** | **Question 10** | **Score** |
| --- | --- | --- | --- | --- | --- | --- | --- | --- | --- | --- | --- | --- |
| Aas et al. | 2021 | Yes | Yes | Yes | Yes | Yes | Yes | Yes | Yes | Yes | Yes | 9 |
| Aas et al. | 2022 | Yes | Yes | Yes | Yes | Yes | Yes | Yes | Yes | Yes | Yes | 10 |
| Alkema et al. | 2024 | Yes | Unclear | Yes | Yes | Yes | Yes | Yes | Yes | Yes | Yes | 9 |
| Arranz et al. | 2018 | Yes | No | Yes | Yes | Yes | No | No | Yes | Yes | Yes | 7 |
| Fisher et al. | 2010 | Yes | Yes | Yes | Yes | Yes | Yes | Yes | Yes | Yes | Yes | 9 |
| Mall et al. | 2020 | Yes | Yes | Yes | Yes | Yes | Yes | Yes | Yes | Yes | Yes | 9 |
| Schalinski et al. | 2019 | No | No | No | Yes | Yes | Unclear | No | Yes | Yes | Yes | 7 |
| Trauelsen et al. | 2015 | Yes | Yes | Yes | Yes | Yes | Yes | Yes | Yes | Yes | Yes | 9 |
| Trotta et al. | 2016 | Yes | Yes | Yes | Yes | Yes | Yes | Yes | Yes | Yes | Yes | 9 |

| **Dose** | **Abajobir et al. (2017)** | **Bentall et al. (2012)** | **Croft et al. (2019)** | **Janssen et al. (2004)** | **Kennedy et al. (2016)** | **Liu et al. (2022)** | **McGrath et al. (2017)** | **Morgan et al. (2014)** |
| --- | --- | --- | --- | --- | --- | --- | --- | --- |
| 1 (95% CI) | 1.69 (1.42-2.02) | 1.66 (1.39-1.98) | 1.66 (1.38-2.01) | 1.68 (1.41-2.01) | 1.68 (1.41-2.01) | 1.68 (1.40-2.03) | 1.68 (1.40-2.03) | 1.73 (1.43-2.10) |
| 2 (95% CI) | 2.51 (1.88-3.36) | 2.44 (1.83-3.25) | 2.45 (1.80-3.34) | 2.49 (1.87-3.33) | 2.50 (1.87-3.34) | 2.50 (1.85-3.39) | 2.50 (1.85-3.39) | 2.52 (1.88-3.38) |
| 3 (95% CI) | 3.03 (2.22-4.14) | 2.94 (2.17-3.98) | 2.94 (2.12-4.08) | 3.00 (2.20-4.09) | 3.02 (2.21-4.12) | 3.01 (2.18-4.15) | 3.01 (2.18-4.15) | 2.95 (2.18-4.00) |
| 4 (95% CI) | 3.23 (2.34-4.45) | 3.14 (2.30-4.28) | 3.12 (2.26-4.31) | 3.20 (2.32-4.40) | 3.22 (2.33-4.44) | 3.19 (2.30-4.41) | 3.19 (2.30-4.41) | 3.10 (2.31-4.18) |
| 5 (95% CI) | 3.36 (2.29- 4.93) | 3.29 (2.27- 4.76) | 3.25 (2.25- 4.68) | 3.33 (2.28-4.87) | 3.36 (2.28-4.94) | 3.31 (2.27- 4.81) | 3.31 (2.27- 4.81) | 3.20 (2.30- 4.55) |
| 6 (95% CI) | 3.50 (2.16- 5.67) | 3.44 (2.16- 5.49) | 3.38 (2.15- 5.31) | 3.47 (2.16- 5.59) | 3.50 (2.16- 5.69) | 3.43 (2.15- 5.47) | 3.43 (2.15- 5.47) | 3.30 (2.21- 4.91) |
| 7 (95% CI) | 3.64 (2.00-6.65) | 3.61 (2.01-6.45) | 3.51 (2.01-6.16) | 3.62 (2.00-6.54) | 3.65 (2.00-6.68) | 3.56 (2.00-6.35) | 3.56 (2.00-6.53) | 3.40 (2.09-5.54) |

Supplementary Material Five- One Study Removed Analysis (I)

Supplementary Material Five- One Study Removed Analysis (II)

| **Dose** | **Shevlin et al. (2007)** | **Shevlin et al. (2010)** | **Whitfield et al. (2005)** | **Aas et al. (2021)** | **Aas et al. (2022)** | **Alkema et al. (2024)** | **Arranz et al. (2018)** | **Fisher et al. (2010)** |
| --- | --- | --- | --- | --- | --- | --- | --- | --- |
| 1 (95% CI) | 1.72 (1.38-2.13) | 1.66 (1.39-1.98) | 1.76 (1.39-2.22) | 1.66 (1.39-1.99) | 1.65 (1.40-1.96) | 1.80 (1.56-2.08) | 1.67 (1.41-1.99) | 1.70 (1.42-2.03) |
| 2 (95% CI) | 2.49 (1.79-3.46) | 2.45 (1.83-3.27) | 2.65 (2.02-3.48) | 2.46 (1.84-3.29) | 2.44 (1.84-3.22) | 2.78 (2.18-3.55) | 2.48 (1.86-3.30) | 2.53 (1.89-3.39) |
| 3 (95% CI) | 3.94 (2.09-4.14) | 2.95 (2.17- 4.02) | 3.61 (2.87-4.53) | 2.98 (2.18-4.08) | 2.97 (2.18-4.03) | 3.31 (2.50-4.38) | 3.00 (2.20-4.09) | 3.05 (2.23-4.17) |
| 4 (95% CI) | 3.13 (2.22-4.42) | 3.16 (2.30- 4.34) | 4.82 (3.68-6.33) | 3.20 (2.30-4.46) | 3.22 (2.31-4.51) | 3.37 (2.50-4.54) | 3.22 (2.32-4.46) | 3.23 (2.35-4.46) |
| 5 (95% CI) | 3.28 (2.21- 4.86) | 3.31 (2.27- 4.84) | 6.46 (4.37-9.53) | 3.38 (2.27- 5.03) | 3.43 (2.28-5.18) | 3.33 (2.38-4.66) | 3.38 (2.28-5.01) | 3.35 (2.29-4.91) |
| 6 (95% CI) | 3.43 (2.12- 5.57) | 3.48 (2.16-5.61) | N/A | 3.56 (2.16- 5.88) | 3.66 (2.18-6.15) | 3.30 (2.23-4.88) | 3.55 (2.16-5.83) | 3.48 (2.16-5.61) |
| 7 (95% CI) | 3.60 (1.99-6.51) | 3.65 (2.01- 6.61) | N/A | 3.75 (2.01-7.01) | 3.90 (2.05-7.41) | 3.27 (2.07-5.16) | 3.73 (2.01-6.92) | 3.61 (1.99-6.53) |

Supplementary Material Five- One Study Removed Analysis (III)

| **Dose** | **Mall et al. (2020)** | **Schalinksi et al. (2019)** | **Trauelsen et al. (2015)** | **Trotta et al. (2016)** |
| --- | --- | --- | --- | --- |
| 1 (95% CI) | 1.71 (1.43-2.04) | 1.71 (1.42-2.06) | 1.73 (1.44-2.10) | 1.69 (1.41-2.02) |
| 2 (95% CI) | 2.56 (1.91-3.44) | 2.57 (1.89-3.50) | 2.53 (1.89-3.38) | 2.52 (1.88-3.38) |
| 3 (95% CI) | 3.08 (2.25-4.22) | 3.11 (2.23-4.35) | 2.99 (2.20-4.05) | 3.04 (2.22-4.16) |
| 4 (95% CI) | 3.25 (2.35-4.49) | 3.32 (2.33-4.72) | 3.17 (2.32- 4.33) | 3.24 (2.34-4.47) |
| 5 (95% CI) | 3.35 (2.29-4.91) | 3.46 (2.27-5.26) | 3.30 (2.29- 4.75) | 3.37 (2.29-4.96) |
| 6 (95% CI) | 3.46 (2.15-5.55) | 3.60 (2.14-6.08) | 3.44 (2.19-5.39) | 3.52 (2.16-5.71) |
| 7 (95% CI) | 3.69 (1.97-6.93) | 3.89 (1.95-7.77) | 3.57 (2.00-6.38) | 3.80 (1.99-7.24) |

Supplementary Material Six- Sensitivity Analyses, Odds Ratios and 95% Confidence Intervals without pooled data

| Number of Cumulative Traumatic Events Reported | OR | 95% CI |
| --- | --- | --- |
| 0 | 1.00 | (1.00-1.00) |
| 1 | 1.89 | (1.55-2.29) |
| 2 | 2.85 | (2.31-3.53) |
| 3 | 3.73 | (3.07-4.54) |
| 4 | 4.76 | (3.51-6.46) |
| 5+ | 6.07 | (3.80-9.68) |
